# High-Throughput and Memory-Efficient Pipeline Key–Value Store Architecture on FPGA

**DOI:** 10.3390/mi16121398

**Published:** 2025-12-11

**Authors:** Xinshuo Wang, Lei Liu, Yifei Li

**Affiliations:** 1 National Network New Media Engineering Research Center, Institute of Acoustics, Chinese Academy of Sciences, No. 21, North Fourth Ring Road, Haidian District, Beijing 100190, China; wangxs@dsp.ac.cn (X.W.); liyf@dsp.ac.cn (Y.L.); 2 University of Chinese Academy of Sciences, No. 19(A), Yuquan Road, Shijingshan District, Beijing 100049, China

**Keywords:** KVS, hardware acceleration, FPGA, parallel pipelined

## Abstract

The increasing speed of network connections is placing increasing demands on the performance of network security and monitoring systems, where Key–Value Stores (KVSs) are becoming critical in network security applications. There is a compelling demand to enhance both the throughput and storage utilization of KVSs. The FPGA-based parallel architecture presents a remarkable opportunity to achieve outstanding performance and power efficiency. In this paper, we propose an FPGA-based implementation of KVSs using a multi-level multi-hash approach, which can effectively avoid false misses and false inserts, in addition to addressing skewed workloads. Decoupled storage exceeds 95% memory utilization, and the pipeline scheme achieves high performance, reaching 400 million requests per second (MRPS). The latency of insert, query, and delete operations is only 60 ns.

## 1. Introduction

With the rapid development of Internet technology, the number and scale of network connections continue to grow. It is expected that network traffic will continue to grow at an explosive rate in the future. According to annual reports presented by Telefónica, global Internet traffic demand is expected to remain at 30% annual growth until 2030 [1]. Therefore, it is becoming increasingly important to handle large volumes of network traffic.

Considering the requirements for high throughput, low processing latency, and operational flexibility, the use of field-programmable gate arrays (FPGAs) as a processing platform in network applications is suitable [2,3]. The high concurrency and pipelining mechanisms of this technology make it ideal for various network applications, such as packet classification [4,5,6,7], traffic monitoring [8,9,10], and packet matching [11,12,13]. Accurate network traffic statistics, packet forwarding and filtering, and security tracking rely heavily on fast lookup algorithms for flow identification. Key–value search is a fundamental operation in data processing and is widely used in various network applications. KVS, which is implemented based on hash algorithms and their derivatives, is popular due to its efficient search capability, ease of implementation, and scalability. In addition, KVS is essential for many high-throughput applications as it provides efficient storage and retrieval for large-scale datasets.

According to [14,15,16,17,18,19], several hardware architectures have been developed to accelerate the processing of KVS. Most of the proposed solutions aim to optimize storage utilization by means of implementing one or multiple parallel instances of the cuckoo hashing [20] technique on FPGA platforms. It is well established that the cuckoo hash architecture achieves remarkable efficiency in software applications; however, with respect to its implementation on FPGA, some challenges still remain unresolved:

- **False misses (insert/query)**: When a key is removed from its original bucket but has not yet been placed into its alternate location, it becomes temporarily unavailable and cannot be reached from either of the buckets. If the insert request is not atomic (i.e., cannot be completed within a single clock cycle), query requests may potentially return a false miss result by concluding the query during a period where the key is temporarily unavailable.
- **False inserts (insert/insert)**: When two consecutive insert requests with conflicting hash addresses arrive at the same empty bucket, the first key–value pair (KVP) will be successfully inserted. However, if the second request cannot promptly retrieve the occupancy status of the bucket due to the read and write-back delays of the BRAM (which require at least three clock cycles), it may overwrite the successful insertion of the first request, resulting in a false insert.
- **Skewed Workloads**: Cuckoo hashing [20] guarantees constant query and insert times, even in the worst-case scenario. In software design, serializing and locking insertion requests is a common strategy to prevent deadlocking [21]. However, in FPGA implementations, using serialized blocking can lead to considerable performance degradation, particularly under high load rates where hundreds of clock cycles may be required to complete a single insert request. Meanwhile, concurrent insertions carry the risk of deadlocks, thus posing a challenging trade-off in the implementation of cuckoo hashing on FPGA.

In this paper, we present a redesigned architecture for the Key–Value Store that effectively mitigates the critical issues previously discussed. This redesigned architecture is capable of achieving high throughput and storage utilization rates while maintaining pipeline processing capability for query and insert requests under various workloads. The main contributions of this report are as follows:

1. To address the issues of false misses and false inserts, we propose a multi-pipeline architecture and integrate the conflict detection module after each level of the pipeline to ensure strict eventual consistency.
2. Inspired by [14], we design a Content-Addressable Memory (CAM) block at the end of the pipeline to improve the robustness of our architecture. Moreover, this design enables effective parameterization and scalability while maintaining excellent timing closure.
3. To ensure the high performance of our KVS architecture under all workloads, we chose the multi-level multi-hash approach and provide mathematically derived calculations of the hash collision probability for this method. Our decision is supported by rigorous software and hardware algorithmic simulations. Finally, our new architecture is successfully implemented on FPGA. Even at a load factor of 95%, the receivable throughput for all types of requests can still reach 400 million requests per second (MRPS), which is 2x faster than [15].

The remainder of this article is organized as follows: Section 2 introduces the related work for this paper, including KVS in FPGA, cuckoo hash, and parallel hash. Section 3 presents our abstract model of the request process, encompassing both a probe phase and a response phase. Section 4 discusses the parallel implementation details of this architecture and an analysis of the improvements achieved through parallelization. Section 5 provides mathematical calculations and analyses of the storage load factor and collision probability for the multi-level multi-hash methodology. Section 6 presents experimental details and compares the results with existing methods. Finally, we conclude the paper in Section 7.

## 2. Related Work

### 2.1. KVS in FPGA

The continued increase in speed and complexity of network devices has resulted in a demand for higher-throughput exact matching in various applications, including intrusion detection system (IDS) pattern matching tables [15,22], flow caching [23], routing lookup [24], stateful network functions [25,26], etc.

Request types comprise insert, delete, modify, and query. Most applications generate frequent query and modify requests, with only a small number of insert and delete requests. However, there are exceptions, such as applications like pattern matching tables for IDSs and flow caching. These applications generate a large number of insert and delete requests to the KVS due to frequent flow switching and the presence of a large number of mice flows in the network.

In network application scenarios, KVS has specific requirements. If the insert performance of KVS is limited, it may become a performance bottleneck for network applications. The rapid growth of network transmission bandwidth has resulted in the need for line-rate or bubble-free pipelining in most network applications. If KVS cannot guarantee data consistency, it may result in increased network burden, such as matching the hash table when forwarding routing and causing false misses, which generates Packet-In messages and requires the network controller to redistribute the flow table. Furthermore, it may lead to network security vulnerabilities, such as false inserts in the network blacklist of the firewall [27], allowing banned IPs to still access protected network devices. In contrast to KVS [28] used in Artificial Intelligence (AI) applications, KVS used in network scenarios demands high performance and strict guarantees of data consistency.

### 2.2. Cuckoo Hash

**Figure 1:**
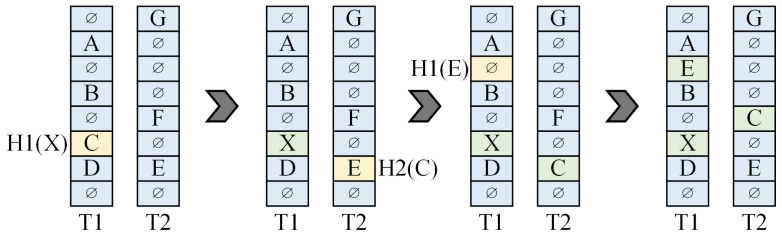
Cuckoo hashing work mechanism.

Cuckoo hashing is an effective method for achieving exact matching in certain applications. It provides good memory utilization and deterministic worst-case access time [16]. Refs. [16,18,29,30] improve the memory utilization of KVS systems by implementing cuckoo hash on FPGAs. Figure 1 shows that cuckoo hashing aims to solve the problem of low memory utilization caused by hash collisions through eviction strategies. However, this approach also has two negative effects.

Firstly, the insert operation of the cuckoo hash cannot be executed concurrently. An insert request may modify a set of buckets when moving the keys along the cuckoo path until one key lands in an available bucket. It is not known before swapping the keys how many times the keys are used and which buckets will be modified, because each displaced key depends on the one previously kicked out. Only when one insert request is fully executed can the second insert request continue.

Secondly, when implemented in an FPGA, the more kick-outs that occur during hash insert, the lower the overall throughput. For example, a cuckoo hash KVS system working at 200 MHz sets an eviction number to 100, a common value. However, under a high load factor, the insert frequency for this KVS system will decrease to approximately 2 MHz, which is unacceptable for applications such as intrusion detection systems and flow caching that require frequent replacement of entries.

### 2.3. Parallel Hash

**Figure 2:**
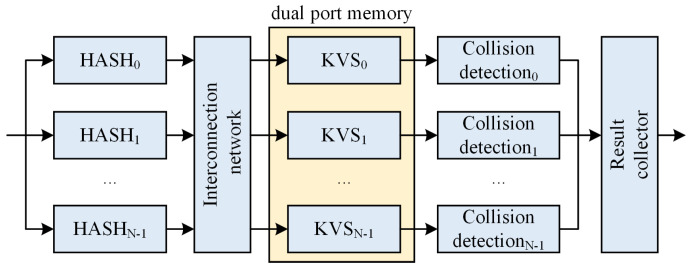
Parallel hash architecture in [15].

As shown in Figure 2, ref. [15] uses 128 parallel different hash functions on an FPGA to build a pattern set for IDS applications. It accepts a request within one cycle and accesses 128 hash tables simultaneously for exact pattern matching of short strings. The advantage of the architecture proposed in [15] is that its 128 parallel hashes increase the storage load rate to about 95%, enabling it to handle various workloads and making it well-suited for IDSs. However, this architecture has relatively low scalability, and achieving full parallelism may be challenging when comparing all output responses with fewer logical resources while maintaining excellent timing closure. Additionally, it is difficult to ensure the strict eventual consistency of the data, which may lead to the generation of false positives or false negatives that compromise network security.

**Figure 3:**
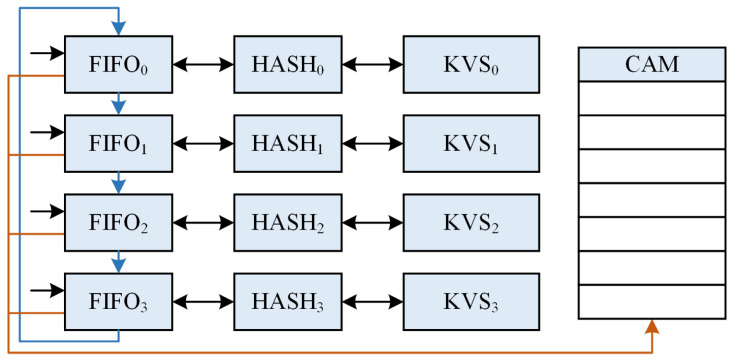
Parallel hash architecture in [17].

As shown in Figure 3, ref. [17] refers to [16], which employs a four-way parallel hash to construct a KVS. Ref. [17] can handle up to four requests per cycle, with each request accessing a distinct hash table. The response is generated only after a successful key match. If a request fails, it moves on to the next hash table. As a result, the subsequent hash table will be unable to accept external requests in the following cycle. This scheme guarantees minimum performance equal to the frequency of the system clock. However, a small number of parallel hash tables cannot guarantee a high storage load factor. If the parallel hash tables of this scheme are increased to improve the load factor, keeping the same size as [15], the system requires more tables. For instance, 128 hash tables are implemented to achieve a 95% load factor. In the worst case, this scheme will traverse these 128 hash tables serially, and 128-deep response reordering needs to be performed. The overall latency would be about 1 to 2 μs, while a layer 3 switch would have a latency of about 2 μs for forwarding 64B packets. This results in almost double the latency metrics of the network device.

## 3. Request Abstraction

When defining a function with KVS functionality, it is customary to equip it with the capacity to handle four distinct types of requests: insert requests, delete requests, modify requests, and query requests, as shown in Table 1. We can categorize the processing of requests into two primary phases: a probe phase and a response phase.

By summarizing and merging similar processes utilized during the handling of these requests, it is feasible to optimize and pipeline the hardware structure design effectively. Hence, such an approach can have significant implications in the development and implementation of efficient and well-functioning hardware systems.

### 3.1. Probe Phase

Regarding the probing phase, our module needs to map the request to an address in the memory module for probing based on the key in the request. It should then determine the relationship between the queried key and the key in the request, and if it meets the conditions of the action execution, it is considered a successful probe. Otherwise, it is considered a failed probe.

The most commonly used methods for calculating address mapping are hash computation, content comparison, and random methods. The address mapping methods used in our architecture are hash and content comparison methods. They correspond to the hash table and CAM, respectively.

### 3.2. Response Phase

Regarding the response phase, our module is responsible for executing the actions specified in the request. If the probing phase yields a successful query, the requested action should be executed, and a response result provided. The type of action is listed in Table 1. If the probing phase yields a failed query, the requested action will not be executed, and a failure response will be provided. Noteworthy details regarding the execution of each action in the response phase will be noted later.

(1) For an insert request, we must verify if the key–value mapping retrieved from the memory module is empty, for which we allocate a bit in the entry to indicate its validity. If it is empty, we write this entry back to the corresponding memory.

(2) For a delete request, we first verify if the key in the entry matches the key in the request. If there is a match, we reset the valid bit and write the updated entry back to the corresponding memory.

(3) For a modify request, a similar approach is taken where we verify whether the key in the request matches the key in the entry. If it does, we perform the requested action (e.g., self-added or self-subtracted, etc.) on the value in the entry. Finally, we write the result back to the corresponding memory and provide the original value in the response.

(4) A query request is simpler to handle, as we only need to verify if the key in the entry matches the key in the request; if it does, we generate the corresponding value response without the need to write any updates back.

### 3.3. Request and Response Bus

The proposed architecture is based on a pipeline implementation, as shown in Figure 4. In the case of a pipeline level located in the middle, if the previous level provides a successful response, this level needs to pass the response to the next level. If the previous level provides a failed response, this level identifies the response as a request and processes it normally. So the request and response bus signals should be the same.

The request/response bus should include the following signals: KEY, VALUE, OP_CODE, OP_NUM, HIT, and VALID.

KEY is used to identify the data.

VALUE is the actual data being stored.

OP_CODE is used to identify the type of request, including insert, delete, modify (self-added or self-subtracted), and query.

OP_NUM indicates the operand and is only used in combination with OP_CODE in modify requests. It atomically updates the VALUE using OP_CODE on scalar OP_NUM. For instance, in the SDN counter application, to count the number of packets in a data flow, set the OP_CODE to self-added and the OP_NUM to one. When a request matches an entry, the VALUE of that entry is incremented by one. Similarly, to count the amount of traffic in a data flow, set the OP_CODE to self-added and the OP_NUM to the packet length.

HIT is used to determine whether the request has successfully matched an entry. If the previous pipeline sends an HIT signal that has been asserted, it indicates that the request has been successfully processed and the current pipeline can simply transfer the response.

The VALID signal, taken from the AXI4-Stream protocol [31], indicates to the slave that data has been presented on the bus when VALID is asserted. The signal indicates that the current data on the bus is valid and can be processed.

## 4. Proposed Architecture

**Figure 4:**
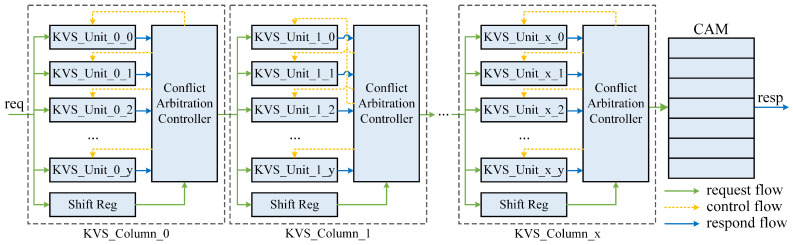
Multi-pipeline KVS matrix architecture.

We propose a multi-level pipelined KVS architecture, as depicted in Figure 4. Our architecture is represented by a matrix with multiple KVS Units arranged in columns. This design enables effective parameterization and scalability while maintaining excellent timing closure.

### 4.1. KVS Unit

**Figure 5:**
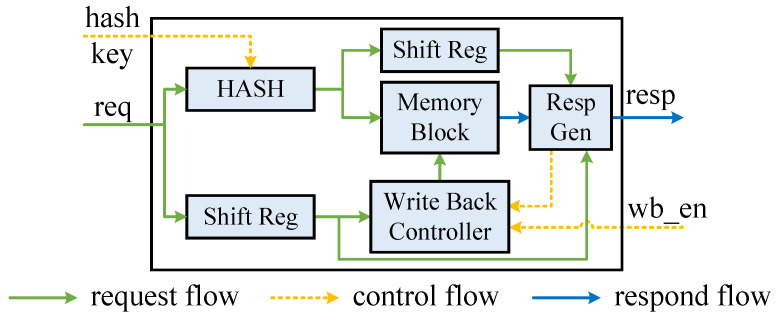
KVS Unit {req & resp: key, value, op_code, op_num, hit, valid}.

Based on the described KVS module workflow, we can define the requisite interfaces for the module. As shown in Figure 5, the KVS Unit necessitates interfaces enabling request reception, response generation, hash key update, and entry write-back functionalities.

The KVS Unit is internally pipelined. Its workflow includes accessing the request, calculating the hash address, reading the entry from memory, and making the appropriate decision based on the type of request.

In our design, each KVS Unit has its own unique hash function. What is more, each KVS Unit can independently handle delete, modify, and query requests for table entries. These requests can be targeted to a single entry by a unique key, even though we have many parallel KVS Units. Therefore, only one of the many KVS Units will be selected to perform the action for these categories of requests. Insert requests require a more complex process. For an entry to be inserted, the entry actually has the chance to be placed in any KVS Unit. However, a single KVS Unit cannot decide whether it can handle the insert request or not. It must communicate and negotiate with other KVS Units in the same pipeline level to elect a slot capable of performing the insertion action. The election process and consistency guarantee are described in Section 4.2.

Continuously incoming delete, modify, and query requests must be consistent in the KVS Unit. However, unlike implementations of hash tables in the popular direction of AI application acceleration, AI applications are approximate in nature and can tolerate small errors in observations or computations [28]. Therefore, the semantics of relaxed eventual consistency are allowed. By contrast, this would be unacceptable in a network application, where relaxed consistency would have serious consequences. For example, it could cause the flow state transition to fail, resulting in a semi-connected state of the data flow and leading to the emergence of network security threats. Thus, we have designed the processing logic of these requests carefully to ensure strict eventual consistency.

As there is a latency in reading data from the BRAM and writing back the modified data, typically 2∼3 clock cycles, incidentally, a longer latency can lead to easier timing closure and a higher clock frequency on the FPGA. One of the most complex processing scenarios is the arrival of continuous modify requests with the same key. This is because a modify operation requires first reading the corresponding entry from the memory, then performing scalar operations on the read value, and finally writing it back to the memory. Subsequent modify requests that come immediately after will read the old value from the memory. Without additional processing, it is possible that only the last modify request produces an actual effect, and the previous requests are executed spuriously. This could have negative consequences for certain network security applications, such as hardware network firewall failures and misaligned data flow states.

So, we take the modify request as an example and design the processing flow as shown in Algorithm 1. During the data writing back latency cycles, the KVS Unit caches the previously entered request using the shift register illustrated in Figure 5. When the entry is read out from BRAM, match the key first. Before executing the requested operation, it is necessary to determine whether any request has performed an operation on the same entry during the previous write-back latency cycles. If there are corresponding in-flight operations, the requested operation is first applied to the last corresponding in-flight operation; subsequently, the final result is written back to the BRAM, and the original computed value to be responded to is returned.

Delete and modify requests are decided and written back by the KVS Unit alone, while insert requests are decided and written back by the KVS Column. To avoid port contentions, the opportunity of the write-back enable signal must be controlled. After receiving a response from the KVS Unit, the KVS Column takes one clock cycle to determine an available slot, ensuring good timing closure. To prevent conflicting write-back signal events, it is recommended to delay the write-back enable signal from the Resp Gen module in the KVS Unit by one cycle and send it to the write-back controller module. This allows the two write-back signals to be sequentially written back to the memory.

Other combinations of requests that arrive consecutively and involve the same entry should be treated similarly. The main idea is to use shift registers to cache intermediate processes that may occur during the latency period. After the entry is read out, the action process of the previous request is re-imposed, and the action of the latest request is executed on this basis.

In addition, through actual experimentation conducted on the FPGA platform, we performed practical evaluations of various hash functions [32,33,34] in terms of their resource utilization, timing closure, and computational latency. After precise comparison of all relevant performance metrics, we ultimately opted for the H3 hash function [32] as the preferred choice for implementing the hashing algorithm within the KVS Unit. The H3 hash seed matrix required by the hardware is randomly generated by software, converted to Verilog, and hardened in the hardware. After the H3 hash matrix is generated based on random numbers, to ensure the uniformity of the hash algorithm, non-compliant H3 matrices must be eliminated in accordance with the following four criteria: (1). Any two rows are distinct. (2). No row is all zeros. (3). After expanding the matrix elements into binary form, no column is all zeros. (4). After expanding the matrix elements into binary form, any two columns are distinct.

Note that the idea of *double hashing* [35] is used in our architecture to solve the problem of hash collisions, as explained in Section 5. Therefore, each KVS Unit has a unique hash function to ensure uniform and independent hashes.

One limitation with FPGA memories is that they only have two ports. Furthermore, true dual-port BRAMs (two simultaneous read/write ports) require double the resources of simple dual-port BRAMs (one read port and one write port). To improve resource utilization efficiency, we opted for simple dual-port BRAM. To ensure that incoming requests are handled at line rate and to avoid port contention, we delegate all write-back tasks to the write-back controller, thus exclusively monopolizing the write port while requests occupy the read port.


**Algorithm 1:** Modify request processing flow in KVS Units.

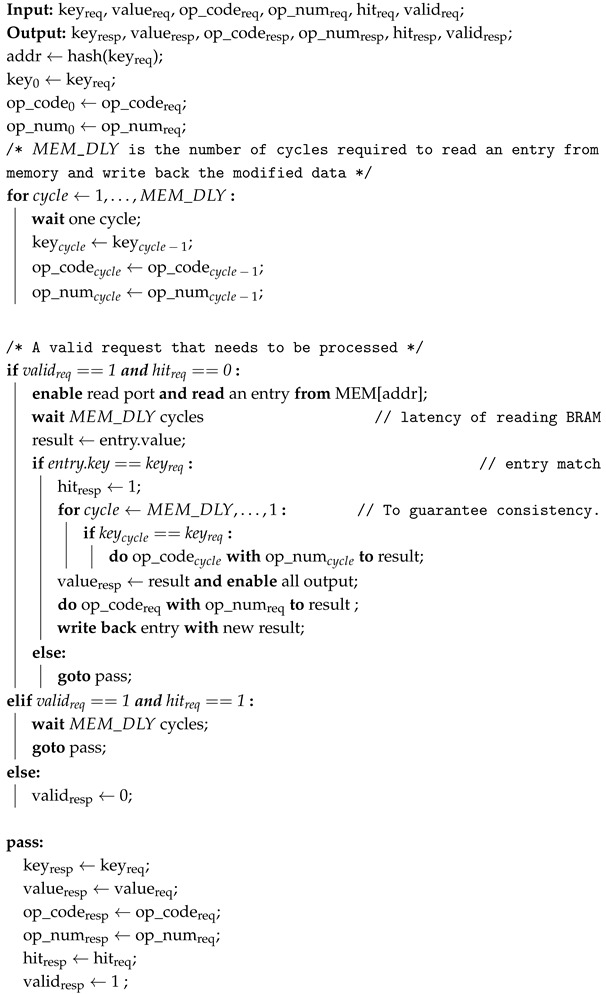




### 4.2. KVS Column

Each KVS Column is composed of multiple KVS Units, the number of which can be passed in by the top-level parameters. When a request arrives, each KVS Unit undergoes a probe phase to read the corresponding entry. Next comes the response phase, during which the KVS Unit can generate and provide the final response if it is not a insert request. For insert requests, KVS Units will, respectively, determine whether the address is empty and whether the key of the entry matches that of the request, thereby providing the corresponding response.

The KVS Column arbitrates among the responses from the KVS Units and then generates the response/request to be delivered. For delete, modify, and query requests, at most one KVS Unit can provide a valid response, which KVS Column simply identifies as a successful request and proceeds to pass it down. Otherwise, the original request will be passed on. For insert requests, there may be multiple KVS Units that produce valid responses. In such cases, the KVS Column needs to arbitrate among them and select one KVS Unit to provide a write-back signal while discarding the other responses.

**Figure 6:**
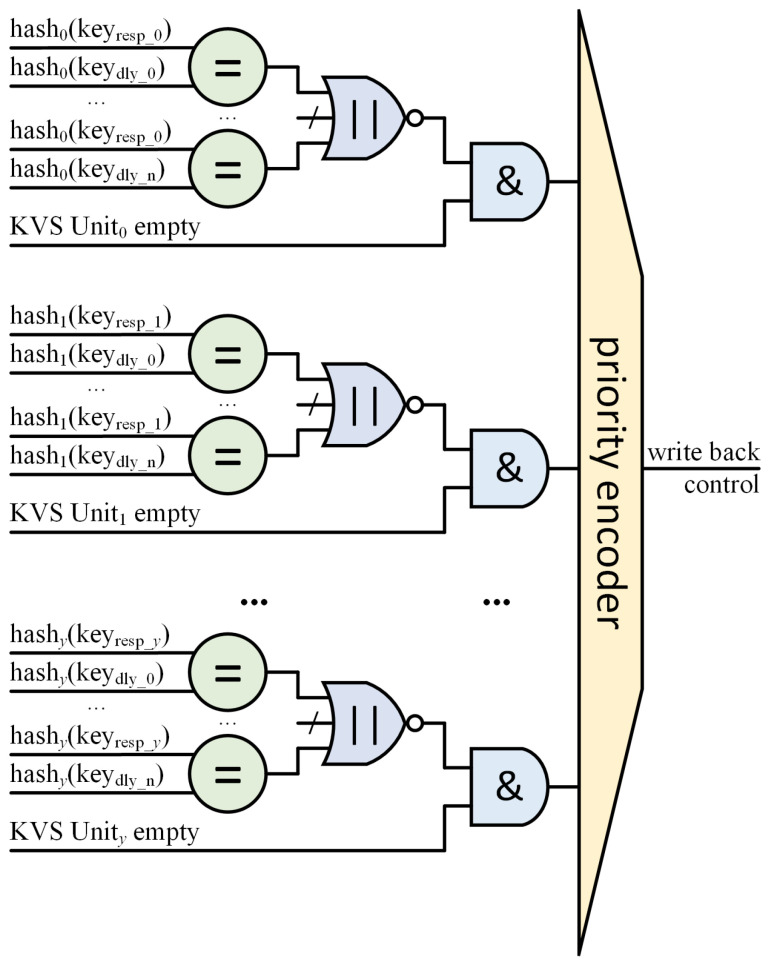
Hash slot election circuit in conflict arbitration controller. (The equal sign (=) represents a comparator. The logical or sign (‖) represents an OR gate. The and sign (&) represents an AND gate. n = *MEMDLY-1*).

This requires the design of a conflict arbitration controller (CAC) module for insert requests in the KVS Column. The CAC module also needs to ensure the consistency of insert requests to prevent false inserts. The circuit schematic of our designed CAC module is shown in Figure 6. The CAC is functionally a priority encoder which has *y* inputs, and *y* is the number of KVS Units in one KVS Column. For an insert request, the CAC receives responses from each KVS Unit as to whether the address where the insert request is to be placed is available. However, this information alone does not ensure data consistency.

Since there is a latency in reading data out of memory and writing it back in, this may cause the current empty state from the KVS Unit to be false. Assuming that two adjacent requests in the same KVS Unit happen to want to insert the same address, the second request will not be able to see the first request’s insert data. The consequence is that a false insert is generated and the first insert request is overwritten by the second request. This is a probabilistic event that occurs due to hash collisions.

So, we use shift registers to save the insert request addresses (hash(key_dly_)) during the write-back latency. Each insertion address is compared one by one to determine if there are any conflicts with previously arrived requests. Only if the address of the insert request is available (empty) and (AND gate in Figure 6) does not conflict with any of the insert request addresses during the write-back latency (NOR gate in Figure 6) is it considered to be a KVS Unit that can respond to that insert request. Although the judgment condition may appear strict, our implementation utilizes 128 KVS Units, each with its own independent hash function. This provides 128 opportunities to solve collisions for insert requests while still maintaining a high overall load factor.

### 4.3. KVS Matrix

The KVS matrix comprises at least one KVS Column and a CAM. The number of KVS Columns can be passed in by the top-level parameter. Each KVS Column has the same function. The CAM is located behind the last KVS Column in the KVS matrix. The CAM is responsible for storing the requests that cannot be processed by all the previous KVS Columns. It is inspired by [14] for improving the KVS system’s robustness.

When a request is received, if the current KVS Column obtains a successful response, it encapsulates the responding KVP in the request for the next KVS Column (if any) and indicates that the request has been responded to. If the current KVS Column does not provide a successful response, the request is passed down to the next KVS Column directly. The subsequent pipeline passes this message downstream until it reaches the matrix’s exit.

If a request cannot find a match in all KVS Units, it would be sent to the CAM for probing. CAM also supports insert, delete, modify, and query requests. Mismatch in all KVS Units and CAM results in the request being failed. Exact matching and writing operations on CAM implemented using registers only use one clock cycle. Thus, the memory access time during the probe phase and response phase is constant, preventing pipeline stalling.

CAMs implemented using registers can be challenging to scale up due to layout routing difficulties. However, we believe that the preceding KVS Column should be able to process the vast majority of requests, and only a few requests cannot be stored by the preceding hash table due to intensive hash collisions. Therefore, a small CAM is placed at the end of the pipeline to resolve hash collisions by storing the KVPs that cannot be inserted into any of the KVS Units. This serves as a final guarantee for the entire KVS system. The idea behind our design is to provide multi-hash sub-tables to deal with collisions. Additionally, a small CAM can handle requests that cannot be inserted into any of the hash tables, resulting in an approximately non-collision hash insert scheme. Details of the theoretical calculations that can resolve most collisions with only a small CAM are shown in Section 5.3.

Our proposed architecture increases the overall storage utilization by constructing a multi-level multi-hash pipeline. Strict eventual consistency is built into each KVS Unit. Due to the absence of an eviction policy, false misses are eliminated, in contrast to cuckoo hashing.

At the heart of our design is to increase the overall load factor as much as possible while maintaining strict eventual consistency. We now argue (Figure 4) why an architecture like the KVS matrix is simple and natural.

To improve the overall load factor and reduce hash collisions, a multi-hash sub-table structure is required for implementing multiple re-hashing operations. When we want to arrange a large number of hash tables, there are two options: vertical and horizontal arrangement.

Vertical arrangement: Arranging a large number of hash tables vertically, i.e., placing a large number of KVS Units (e.g., 128, 256, etc.) in a KVS Column, can make timing closure difficult, as the decision to insert slots has to be made quickly (within one clock cycle) to ensure data consistency.

Horizontal arrangement: Horizontal arrangement of all KVS Units, on the other hand, does not result in data inconsistency issues. However, this will generate a very long pipeline (e.g., 128 levels and each level generating 3∼4 cycles of latency) and result in microsecond-level latency just for querying the table. This seems to be unacceptable in a network application. After measuring, we found that the latency for forwarding a 64 B packet in layer 3 of the H3C commercial switch [36] is approximately 2 μs.

Thus, the KVS matrix architecture arises naturally, combining multiple considerations of timing closure, layout routing, load factor, and path latency. Each KVS Column is pipelined, ensuring that our architecture can process requests on the pipeline with no bubbles and has good scalability. In addition, developers can choose KVS matrix specifications like building blocks to meet their needs.

After implementation experiments, the empirical value recommended is 32 × 4. This means that the KVS matrix has 4 KVS Columns, each with 32 KVS Units. This configuration ensures a high load factor (see Section 5.2 for details) while maintaining great timing closure and latency parameters.

## 5. Hash Table Guarantees

This section presents our approach to resolving hash collisions and its theoretical analysis. Open addressing and separate chaining are the two collision resolution techniques [37]. They play a vital role in the analyses and discussions. The use of separate chaining is not recommended for implementation on FPGA due to the difficulty in parallelizing and pipelining the structure of the linked list, which makes it challenging to achieve a query rate that meets the line rate. So our hash table is implemented based on open addressing techniques.

### 5.1. Open Addressing

Open addressing is a technique where all elements are stored within the hash table itself. During a query, table slots are systematically examined until the desired element is found. Elements are not stored outside the table. Techniques such as linear probing, quadratic probing, and double hashing fall under open addressing. Furthermore, double hashing offers one of the best methods available for open addressing because the permutations produced have many of the characteristics of randomly chosen permutations [35].

Double hashing uses a hash function of the following form:

(1) $$h\left(key,i\right)=(h_{1}\left(key\right)+ih_{2}\left(key\right))\;mod\;m;\;\;i\in \left\{0,1\right\}.$$

Here, $h_{1}$ and $h_{2}$ are auxiliary hash functions, and *m* is the size of the hash table. The initial probe goes to position $Table[h_{1}\left(key\right)]$. Subsequent probe positions are offset from previous positions by the amount $h_{2}\left(key\right)$, modulo *m*. Thus, unlike linear or quadratic probing, the probe sequence here depends on the key in two ways, since the initial probe position, the offset, or both can vary.

In relation to our FPGA implementation, as shown in Figure 4, it is important that the hash function for each hash sub-table is different from one another to prevent clustering, thus aligning with the concept of double hashing. If the hash function for each sub-table is the same, it can lead to primary clustering issues [38], resulting in a higher average number of probing and an increased probability of collisions.

### 5.2. Collision Probability

Given an open-address hash table with load factor $\alpha =\frac{n}{m}<1$, which means there are *n* elements and *m* slots, assuming uniform hashing. In an unsuccessful insert, every probe including the last accesses an occupied slot. The random variable *X* is defined as the number of probes made in an unsuccessful insert, and let us also define the event $A_{i}$ (*i* = 1, 2, ⋯) to be the event that an *i*th probe occurs and it is to an occupied slot. Then, the event {X≥i} is the intersection of events $A_{1}\cap A_{2}\cap \cdots \cap A_{i}$. We will bound Pr{X≥i} by bounding $Pr\{A_{1}\cap A_{2}\cap \cdots \cap A_{i}\}$. By multiplication rule of probability,

(2) $$\begin{array}{c} Pr\left\{A_{1}\cap A_{2}\cap \cdots \cap A_{i}\right\}=Pr\left\{A_{1}\right\}\times Pr\left\{A_{2}|A_{1}\right\}\times \\ Pr\left\{A_{3}|A_{1}\cap A_{2}\right\}\times \cdots \times Pr\left\{A_{i}|A_{1}\cap A_{2}\cdots \cap A_{i-1}\right\}\;. \end{array}$$

Since there are *n* elements and *m* slots, $Pr\left\{A_{1}\right\}=\frac{n}{m}$. For j>1, the probability that there is a *j*th probe and it is to an occupied slot, given that the first j−1 probes were to occupied slots, is $\frac{n-j+1}{m-j+1}$. This probability follows because we would be finding one of the remaining (n−(j−1)) elements in one of the (m−(j−1)) unexamined slots, and by the assumption of uniform hashing, the probability is the ratio of these quantities. Observing that n<m implies that $\frac{n-j}{m-j}\leq \frac{n}{m}$ for all *j* such that 0≤j<m, we have for all *i* such that 1≤i≤m,

(3) $$\begin{array}{cc} Pr\{X\geq i\} & =\frac{n}{m}\times \frac{n-1}{m-1}\times \frac{n-2}{m-2}\cdots \frac{n-i+1}{m-i+1}\\ \; & \leq {\left(\frac{n}{m}\right)}^{i}\\ \; & =\alpha^{i}\;. \end{array}$$

**Figure 7:**
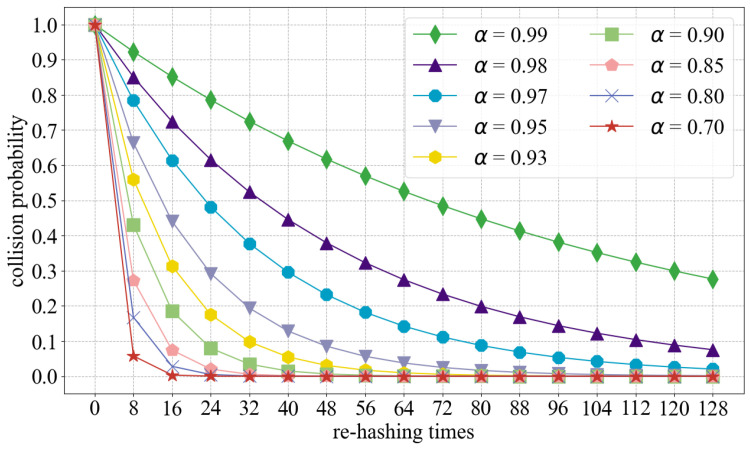
Collision probability.

As the load factor α is less than 1, Equation (3) demonstrates that the probability of a new entry failing to be inserted decreases as the number of re-hashing increases. Figure 7 shows the relationship between the collision probability, re-hashing times, and load factor α. Cases where α is greater than 90% are plotted more as they are more meaningful. Figure 7 indicates that the collision probability decreases relatively slowly when α is 99% or 98%. Even when α = 98% and re-hashing times = 128, there is still a collision probability of about 7.5%. However, when α is less than 95% and the re-hashing times reach 128, the collision probability decreases to the 1‰ level. We also use the specification of 128 re-hashing times in our FPGA implementation to achieve a better hash performance.

### 5.3. Capacity Requirement of CAM

Equation (3) shows that when the load factor is α, the upper bound on the probability of an insert request failing even after re-hashing *i* times to resolve a collision is $\alpha^{i}$. This means that if an insert request probes our KVS matrix *i* times, and each time the result of the probe is occupied, then the request will enter the CAM module for probing. Therefore, the probability that an insert request enters the CAM is $\alpha^{i}$. The CAM capacity $Cap_{CAM}$ can be approximated as the expected number of requests that cannot be inserted into all KVS Units, and the expression for this is as follows:

(4) $$\begin{array}{c} Cap_{CAM}=N\times \alpha^{i}. \end{array}$$

Here, *N* represents the number of requests that are about to enter the KVS matrix. The depth of the hash table in a commercial switch is typically around 64 K [39]. Our recommended number of re-hashing times is 128. Thus, insert requests moving to the CAM are extremely rare even when the load factor is at 95%. At this specification, the required CAM capacity is approximately 65 entries ($Cap_{CAM}=64K\times 0.95\times 0.95^{128}$). This level of CAM depth is relatively easy to implement in FPGAs.

## 6. Experimental Results

In this section, we commence by assessing the feasibility of employing multiple hash sub-tables as the fundamental units of the KVS. Subsequently, we implement a multi-level pipelined KVS architecture on FPGA and evaluate its performance in terms of throughput and memory utilization. Furthermore, a comparison is conducted between the proposed approach and prior FPGA-based implementations of KVS.

### 6.1. Feasibility Analysis

To enhance the utilization of storage space in KVS systems, two common approaches are typically considered: increasing the number of hash tables and utilizing multi-bucket solutions. Each of these methods has its own advantages and disadvantages in software implementation. However, on FPGAs, both schemes consume nearly equal logical and storage resources. The multiple hash table approach involves only a few additional hash address calculations, and both the multiple sub-tables and multiple buckets can be parallelized. Hence, it is imperative to analyze and evaluate the two proposed approaches from a hardware standpoint in order to determine the optimal implementation strategy.

**Figure 8:**
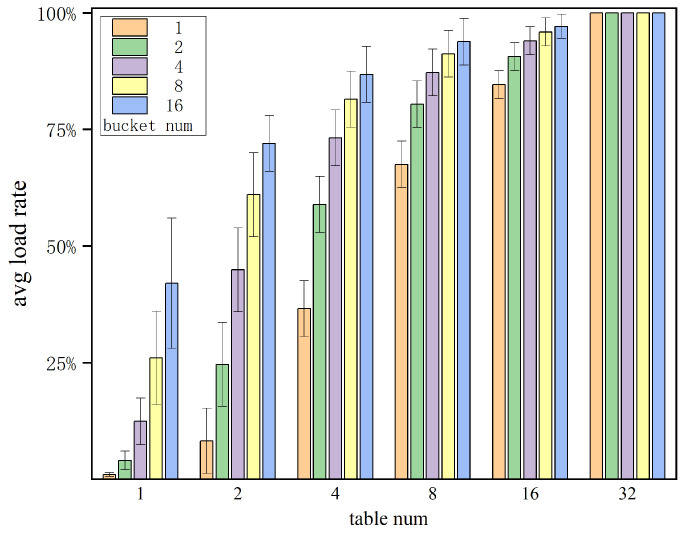
Static hash load rate test.

We conducted simulations using Java to evaluate the behavior of KVS with multiple hash sub-tables and multiple buckets. The simulation process involved randomly inserting entries into 16K storage units until hash collisions became unresolved with the use of multiple cells. We examined different combinations of hash tables and buckets in a variety of storage units, and calculated the storage space utilization, also known as the load factor. Each combination underwent 10,000 trial experiments, and the average values were recorded. As shown in Figure 8, our simulation results indicate that when the number of hash tables and buckets is low, the load factor is also low. However, when the number of hash tables increases to 32, even with only a single bucket per table, the design can achieve a nearly 100% load factor. In contrast, with two hash tables and 16 buckets, the storage space utilization is only about 72%. It is evident that the benefits in terms of space utilization from multiple hash sub-tables are far greater than those from multiple buckets. The use of multiple hashing significantly reduces the probability of entry collisions. For FPGA implementations, where parallelization is feasible, the addition of a small amount of logical resources to calculate multiple hash values yields a significant improvement in storage space utilization. This trade-off proves to be highly advantageous.

Subsequently, we conducted tests on dynamic insertion of KVS entries to determine the relevant parameters for FPGA implementation. This behavior was simulated using Java as well. We instantiated a KVS object with 64 K storage cells and organized them using different numbers of hash sub-tables. We then dynamically inserted 10 M KVPs into the KVS object and periodically performed random deletions of some entries to simulate aging behavior and maintain a constant storage occupancy rate. During the statistical process, we utilized stash space [14], which corresponds to the hardware requirements for Content-Addressable Memory (CAM). However, due to the challenge of efficient CAM implementation on FPGAs [40], we did not track requirements beyond 1024.

**Figure 9:**
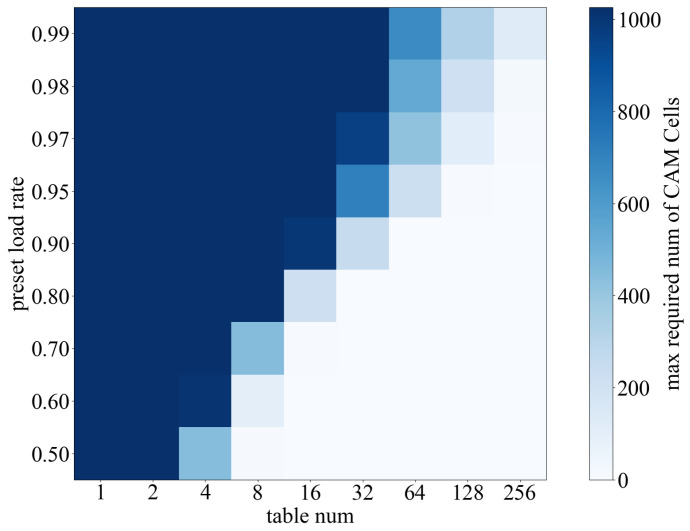
Dynamic hash load rate test.

As depicted in Figure 9, our experimental results show a significant improvement in load factor when the number of sub-tables reaches 128 or higher.

Additionally, the increase in load factor further enables a substantial reduction in the depth of the CAM table, consequently leading to an improvement in the overall system clock frequency. This finding additionally confirms the feasibility of employing multi-hash sub-tables as the fundamental units of the KVS.

### 6.2. Implementation on FPGA

**Figure 10:**
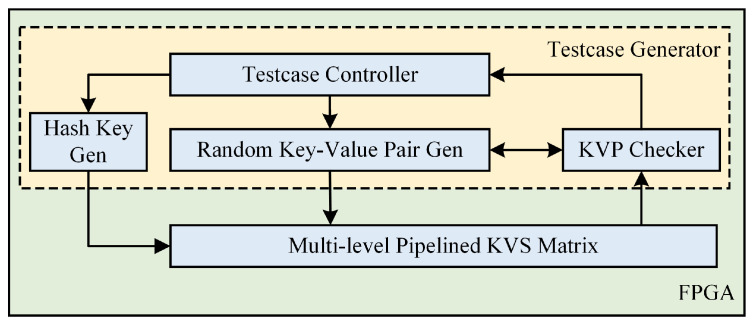
The evaluation platform of the KVS.

In order to assess the proposed multi-level pipelined KVS architecture, we have devised a platform as depicted in Figure 10. The hash key generator module is utilized to inject random hash keys into all hash tables. The random key–value pair generator module is capable of generating pseudo-random data to simulate real-world KVP traffic. The KVP checker module compares the output response results of our architecture with the expected response, thus recording any erroneous outcomes. The testcase controller computes latency and throughput, counts error entries, and transmits the results out of the module for viewing through a register interface or the Vivado ILA tool.

The target FPGA for this implementation is the Xilinx XCVU9P, which provides 2160 BRAM blocks, each with a capacity of 36 Kbits. In our implementation, the key length is 32 bits and the value length is 64 bits. We have a total storage space of 64K entries. The KVS array is instantiated with 32, 64, 128, and 256 KVS Units, with 32 units forming a column. We employ the H3 hash function, which is specifically designed for hardware circuits with low resource consumption. It can calculate the hash result within two clock cycles, ensuring excellent timing closure. Each individual KVS Unit consumes 223 LUTs and 1316 registers of the programmable logic.

The overall resource utilization is shown in Table 2. Without using additional storage using CAM, the design has a load factor of 0.95 when the number of KVS Units reaches 128. In other words, the insertion, query, and deletion operations all require traversing a four-stage pipeline, with a delay of six clock cycles per stage. Therefore, the total latency for a request is 24 clock cycles. An FPGA-based KVS implementation can operate at a frequency of 400 MHz, resulting in a latency of only 60 ns.

Since the base size of a BRAM is 72 bits × 512, there are 64 K table entries, which represents the common depth in flow table design. In the case of a number of sub-tables that is equal to or less than 128, the number of table entries determines the amount of BRAM resources occupied, which is 192 BRAMs. This is because 64 K/128 = 512, which represents the minimum occupied depth of a BRAM. In the event that the number of sub-tables exceeds 128, the number of sub-tables determines the occupation of BRAM resources. This is due to the fact that the depth of sub-tables is insufficient to occupy the entire BRAM. Consequently, for a common flow table depth of 64 K, 128 sub-tables represent the optimal balance between resource utilization and the load factor.

We have implemented our multi-level pipeline KVS architecture on FPGA, and we conducted on-board testing using carefully constructed test cases to evaluate the effectiveness of our system. Additionally, targeted tests were conducted to assess and address the issues of false misses, false inserts, and skewed workloads that were previously raised. The results of our tests demonstrate the successful resolution of the aforementioned challenges by our architecture.

We have achieved full pipeline execution for all operations, where the throughput of each module is equal to the clock frequency of the module. Due to the inherent simplicity in the design of our multi-level pipelined KVS architecture and our optimized implementation, we achieved a sustained operating frequency of 400 MHz without any timing violations. This translates to a throughput of 400 million requests per second (MRPs), which is 2.7x faster than [18] and 2x faster than [15], as demonstrated by the specific comparative experimental results presented in Table 3. By employing slightly more LUT resources, we have achieved an outstanding level of throughput performance and storage load factor.

## 7. Conclusions

In this paper, we have designed a KVS system architecture incorporating multi-hash sub-tables and parallel pipelines, with a comprehensive evaluation conducted on an FPGA platform. Our primary contribution in this paper is the proposal of a KVS system architecture with high throughput and efficient utilization of storage capacity, which can effectively avoid false misses, false inserts, and skewed workloads. Using a multi-level multi-hash structure to reduce the probability of storage hash collisions, this structure naturally supports any workload situation, while also avoiding false misses caused by the eviction operation of cuckoo hashes. Furthermore, a collision detection module is designed within the KVS Unit to avoid false inserts. A small CAM is used to resolve the hash collision by storing the tuples that cannot be inserted to any of the tables.

The experimental results demonstrate that the proposed architecture achieves over a twofold improvement in throughput compared to existing FPGA-based KVS systems. Upon instantiating 128 sub-tables, the system’s load factor can reach 95%, and resource consumption is almost linearly related to the number of KVS Units. Notably, the individual KVS Units exhibit remarkably low LUT resource consumption. These findings confirm the proposed architecture as an effective solution for on-chip FPGA-based KVS systems.

## Figures and Tables

**Table 1:** Request operations.

Function	Explanation
insert(key, value) → bool	Insert a (key, value) pair. Returns true if insertion succeeds and false if the key already exists or all candidate buckets across hash levels are occupied.
delete(key) → bool	Delete a (key, value) pair. Returns true if the key is found in any sub-table or the CAM; returns false otherwise.
modify(key, op_code, op_num) → value	Atomically update the value of key using op_code on scalar op_num, and return the original value. Returns the original value before modification. Guarantees per-key read–modify–write atomicity even under pipeline parallelism and bursty traffic.
query(key) → value	Get the value of key. If found, it returns the stored value; if not found, it returns a special null value.

**Table 2:** Resource consumption of different sub-hash table numbers on FPGA.

Table Num	LUTs (1,182,240)	Registers (2,364,480)	BRAMs (2160)
32	7518 (0.64%)	28,030 (1.19%)	192 (8.89%)
64	14,256 (1.21%)	14,256 (1.21%)	192 (8.89%)
128	27,665 (2.34%)	101,745 (4.30%)	192 (8.89%)
256	53,139 (4.49%)	175,088 (7.40%)	384 (17.78%)

**Table 3:** Comparison with previous methods.

Method	Platform	Clock Frequency (MHz)	LUTs	Throughput (Million Requests/s)	Load Factor
ref. [17], parallel hash *	Xilinx Zynq	301	1653	153.6	0.50
ref. [18], cuckoo hash ^†^	Xilinx Zynq	200	4944	147	0.90
ref. [41], T = 16 ^‡^	Xilinx Zynq	261	7820	62.4	—
ref. [15], CH + B = 8 ^⋆^	Xilinx UltraScale+	200	34,212	200	0.95
Our work ^‡‡^	Xilinx UltraScale+	400	27,665	400	0.95

*: 4-way parallel hash. ^†^: 4-way cuckoo hash. ^‡^: T is parallelism. ^⋆^: CH is the number of cuckoo hash functions; B is the size of buckets. ^‡‡^: 4-level pipeline with 128 hash.

## Data Availability

The raw data supporting the conclusions of this article will be made available by the authors on request.
